# The Knudsen Layer in the Heat Transport Beyond the Fourier Law: Application to the Wave Propagation at Nanoscale

**DOI:** 10.3390/e27111172

**Published:** 2025-11-20

**Authors:** Isabella Carlomagno, Antonio Sellitto

**Affiliations:** Department of Industrial Engineering, University of Salerno, 84084 Fisciano, Italy; icarlomagno@unisa.it

**Keywords:** phonon–wall interactions, nanoscale heat transfer, heat waves, Knudsen layer, 02.30.Jr, 02.90.+p, 05-70.Ln, 44.04.+e

## Abstract

In agreement with the second law of thermodynamics, a new theoretical model for the description of the heat transfer at nanoscale in a rigid body is derived. The model introduces the concept of the Knudsen layer into non-equilibrium thermodynamics in order to better investigate how phonon–boundary scattering may influence the heat propagation at nanoscale. This paper, in particular, deepens the influence of the Knudsen layer on the speed of propagation of thermal waves.

## 1. Introduction

Owing to the continuous search for the advancements of the performances of modern electronic devices, in the design of integrated circuits in particular there are nowadays severe thermal-management challenges; incorrect thermal-management strategies, in fact, may become critical bottlenecks limiting further technological advancements.

Towards the development of correct strategies for the thermal management in modern chips, it is essential to rightly understand how the heat transfer changes in the transition from the macroscale to the nanoscale, wherein it is currently well-known that the classical Fourier law of the heat conduction breaks down [1,2,3,4].

Among the different approaches for the analysis of the heat transfer at nanoscale [5,6,7,8,9], the macroscopic method based on the phonon hydrodynamics [10,11,12,13] is particularly worth considering since it may easily allow us to gain useful information about the physics of the phonons’ motion [5]. In phonon hydrodynamics, in fact, the propagation of the heat flux can be thought of as the consequence of a *regular* motion of the heat carriers across the crystal lattice [10,14]. During that motion the phonons always undergo several scattering mechanisms, that is, the Umklapp phonon–phonon scattering, phonon–impurity scattering, phonon–electron scattering and phonon–boundary scattering [15,16].

The phonon–boundary scattering may have severe consequences on thermal transport in nanostructures [3,10,17] and is particularly relevant in a narrow zone near the system’s boundary whose characteristic dimension is of the same order of magnitude of the mean-free path *ℓ* of the heat carriers [18,19,20,21,22]. For this reason, by taking inspiration from fluid-dynamics, in Ref. [23] it has been introduced for the first time in the analysis of the heat transfer at nanoscale the concept of the Knudsen layer, that is, the portion of the system wherein the scattering of the heat carriers with the system’s boundary prevails among the other scattering mechanisms, and the non-local effects are especially relevant.

In this new hydrodynamical approach to the thermal conductivity at the nanoscale, the Knudsen layer is assumed to be always superposed to the system’s bulk, fully pervading it whenever *ℓ* is larger than the characteristic dimension of the system [23]. The Knudsen layer and the system’s bulk cannot be thought of as separate from each other: it is in fact assumed that a heat carrier always flows in both of them, mainly undergoing the boundary scattering in the Knudsen layer and the other scattering mechanisms (i.e., the Umklapp phonon–phonon scattering, the phonon–impurity scattering and the phonon–electron scattering) in the system’s bulk, which will be therefore named *bulk scatterings* in the next for the sake of simplicity. The theoretical width of the Knudsen layer only turns out information on the relative importance of the phonon–wall scattering with respect to the other scattering mechanisms.

Owing to the theoretical considerations above, in Ref. [23] the authors supposed that the local heat flux $q_{i}$ is always characterized both by a bulk contribution $q_{i}^{b}$, and a wall contribution $q_{i}^{w}$ in such a way that everywhere in the system one has

(1) $$q_{i}=q_{i}^{b}+q_{i}^{w}$$

The bulk heat flux is supposed to be principally related to the bulk scatterings; the wall heat flux, instead, principally arises from the phonon–boundary scattering in the Knudsen layer.

In the present paper we mainly analyze the influence of the Knudsen layer on the propagation of small-amplitude thermal waves. To reach this goal, we here put our attention on the following theoretical model:

(2a) $$\begin{array}{c} \;\;\;\;\;\;\;\;\;\partial_{t}\theta +\frac{q_{i{,}_{i}}^{b}}{c_{v}}=0 \end{array}$$

(2b) $$\begin{array}{c} \partial_{t}q_{i}^{b}+\frac{q_{i}^{b}}{\tau_{b}}+\frac{\lambda }{\tau_{b}}\theta_{{,}_{i}}+Q_{ij{,}_{j}}^{b,0}+Q_{{,}_{i}}^{b}=0 \end{array}$$

(2c) $$\begin{array}{c} \;\;\partial_{t}q_{i}^{w}+\frac{q_{i}^{w}}{\tau_{w}}+Q_{ij{,}_{j}}^{w,0}+Q_{{,}_{i}}^{w}=0 \end{array}$$

(2d) $$\begin{array}{c} \partial_{t}Q_{ij}^{b,0}+\frac{Q_{ij}^{b,0}}{\tau_{b}^{★}}+\frac{2\ell^{2}}{\tau_{b}\tau_{b}^{★}}q_{i{,}_{j}}^{b,\mathrm{sym},0}=0 \end{array}$$

(2e) $$\begin{array}{c} \;\;\partial_{t}Q^{b}+\frac{Q^{b}}{\tau_{b}^{★}}+\frac{5\ell^{2}}{3\tau_{b}\tau_{b}^{★}}q_{i{,}_{i}}^{b}=0 \end{array}$$

(2f) $$\begin{array}{c} \partial_{t}Q_{ij}^{w,0}+\frac{Q_{ij}^{w,0}}{\tau_{w}^{★}}+\frac{2\xi^{2}\ell^{2}}{\tau_{w}\tau_{w}^{★}}q_{i{,}_{j}}^{w,\mathrm{sym},0}=0 \end{array}$$

(2g) $$\begin{array}{c} \;\;\;\;\;\;\partial_{t}Q^{w}+\frac{Q^{w}}{\tau_{w}^{★}}=0 \end{array}$$

wherein θ is the non-equilibrium temperature, $Q^{b}\delta_{ij}$ and $Q_{ij}^{b,0}$ are the spherical and deviatoric parts of the flux of the bulk heat flux $Q_{ij}^{b}$, respectively, whereas $Q^{w}\delta_{ij}$ and $Q_{ij}^{w,0}$ mean the spherical and deviatoric parts of the flux of the wall heat flux $Q_{ij}^{w}$, respectively. In Equation (2a–g), $\tau_{b}$, $\tau_{w}$, $\tau_{b}^{★}$, and $\tau_{w}^{★}$ are the relaxation times of $q_{i}^{b}$, $q_{i}^{w}$, $Q_{ij}^{b}$ and $Q_{ij}^{w}$, respectively. Therein λ is the thermal conductivity, $c_{v}$ is the specific heat per unit volume at constant volume, and $\xi \in \left(0;1\right]$ stands for a non-dimensional parameter which allows us to account for the possible difference of the mean-free-path values of the heat carriers attained in the bulk (i.e., *ℓ* in Equation (2a–g)) and in the Knudsen layer (i.e., the quantity ξℓ in Equation (2a–g)). In the present paper we assume that all aforementioned material functions only vanishingly vary with the temperature, namely, we assume only constant values for them in order to avoid nonlinear terms and therefore reduce our analysis to a simpler level. For this reason, from the very beginning we explicitly state that whenever the thermal-wave amplitudes are not negligible, or in the so-called *very far-from equilibrium situations* (characterized by values of the flux variables which are large enough), nonlinear terms are no longer negligible and Equation (2a–g) should not be employed if one aims at a correct analysis.

We finally note that throughout the paper the superscript “sym0,0” indicates the symmetric and traceless part of the corresponding second-order tensor, whereas the subscript “,*i*” stands for the spatial derivative of the indicated quantity.

As a brief summary, the layout of the present paper is the following. In Section 2 we develop some theoretical considerations in agreement with the second law of thermodynamics which allow us to macroscopically obtain a theoretical model able to account for the Knudsen layer. In Section 3 Equation (2a–g) are straightforwardly carried out by means of some physical considerations about the different coefficients (involved in the aforementioned theoretical model) which are not known a priori. In Section 4 we use Equation (2a–g) to study the propagation of small-amplitude thermal waves. In Section 5 we give final comments.

## 2. Theoretical Considerations: The Model Derivation

Since the model of heat transport in a rigid body described by Equation (2a–g) does not arise either from experimental evidence, or from a rigorous microscopic derivation, we use this section in order to show that it lies, however, on strong theoretical grounds that are in agreement with the second law of thermodynamics.

According with the general tenets of Extended Irreversible Thermodynamics (EIT) [5] we may assume that the state-space S is

(3) $$S=\left\{e;q_{i}^{b};q_{i}^{w};Q_{ij}^{b};Q_{ij}^{w}\right\}$$

wherein *e* is the internal energy density (i.e., per unit volume). As a consequence the generalized Gibbs equation (i.e., the differential of the entropy per unit volume *s*) reads as

(4) $$ds=\frac{\partial s}{\partial e}de+\frac{\partial s}{\partial q_{i}^{b}}dq_{i}^{b}+\frac{\partial s}{\partial q_{i}^{w}}dq_{i}^{w}+\frac{\partial s}{\partial Q_{ij}^{b}}dQ_{ij}^{b}+\frac{\partial s}{\partial Q_{ij}^{w}}dQ_{ij}^{w}$$

wherein $\frac{\partial s}{\partial e}=\frac{1}{\theta }$, as it is usual in non-equilibrium Thermodynamics. In order to carry on with the analysis of the consequences of Equation (4) we may assume the following relations

(5a) $$\begin{array}{c} \frac{\partial s}{\partial q_{i}^{b}}=\alpha_{b}\left(e;q_{i}^{b};q_{i}^{w};Q_{ij}^{b};Q_{ij}^{w}\right)q_{i}^{b} \end{array}$$

(5b) $$\begin{array}{c} \frac{\partial s}{\partial q_{i}^{w}}=\alpha_{w}\left(e;q_{i}^{b};q_{i}^{w};Q_{ij}^{b};Q_{ij}^{w}\right)q_{i}^{w} \end{array}$$

(5c) $$\begin{array}{c} \frac{\partial s}{\partial Q_{ij}^{b}}=\beta_{b}\left(e;q_{i}^{b};q_{i}^{w};Q_{ij}^{b};Q_{ij}^{w}\right)Q_{ij}^{b} \end{array}$$

(5d) $$\begin{array}{c} \frac{\partial s}{\partial Q_{ij}^{w}}=\beta_{w}\left(e;q_{i}^{b};q_{i}^{w};Q_{ij}^{b};Q_{ij}^{w}\right)Q_{ij}^{w} \end{array}$$

wherein $\alpha_{b}$, $\alpha_{w}$, $\beta_{b}$, and $\beta_{w}$ are suitable scalar-valued functions of the indicated arguments. Since at the current state of our analysis their exact physical identifications are not properly needed, we let them for now in a very general form; later on we will relate those functions to well-defined physical quantities. Here, however, we observe that Equation (5a–d) are compatible with the general theorems of representation of the scalar-valued functions that may depend on scalar, vector, and tensor variables [24].

Recalling that the local balance of energy in a rigid body in the absence of heat source yields

(6) $$\partial_{t}e=-q_{i{,}_{i}}^{b}-q_{i{,}_{i}}^{w}$$

once Equation (1) has been taken into account, then from Equations (4) and (5a–d) we are directly lead to

(7) $$\partial_{t}s=-\frac{q_{i{,}_{i}}}{\theta }+\alpha_{b}\partial_{t}q_{i}^{b}+\alpha_{w}\partial_{t}q_{i}^{w}+\beta_{b}\partial_{t}Q_{ij}^{b}+\beta_{w}\partial_{t}Q_{ij}^{w}$$

Under the assumption that the constitutive equation of the entropy flux $J_{i}^{s}$ is

(8) $$J_{i}^{s}=\frac{q_{i}^{b}}{\theta }+\gamma_{b}Q_{ij}^{b}q_{j}^{b}+\gamma_{w}Q_{ij}^{w}q_{j}^{w}$$

with $\gamma^{b}$ and $\gamma^{w}$ being two constant which will be clearly identified from the physical point of view later on, by straightforward calculations Equation (7) can be cast in the classical form of a balance law, i.e., $\partial_{t}s=-J_{i{,}_{i}}^{s}+\sigma^{s}$, if we identify

(9) $$\begin{array}{cc} \sigma^{s}=-\frac{q_{i{,}_{i}}^{w}}{\theta } & +q_{i}^{b}\left(\alpha_{b}\partial_{t}q_{i}^{b}-\frac{\theta_{{,}_{i}}}{\theta^{2}}+\gamma_{b}Q_{ij{,}_{j}}^{b,0}+\gamma_{b}Q_{{,}_{i}}^{b}\right)+q_{i}^{w}\left(\alpha_{w}\partial_{t}q_{i}^{w}+\gamma_{w}Q_{ij{,}_{j}}^{w,0}+\gamma_{w}Q_{{,}_{i}}^{w}\right)\\ \; & +Q_{ij}^{b,0}\left(\beta_{b}\partial_{t}Q_{ij}^{b,0}+\gamma_{b}q_{i{,}_{j}}^{b,\mathrm{sym},0}\right)+Q_{ij}^{w,0}\left(\beta_{w}\partial_{t}Q_{ij}^{w,0}+\gamma_{w}q_{i{,}_{j}}^{w,\mathrm{sym},0}\right)\\ \; & +Q^{b}\left(\beta_{b}\partial_{t}Q^{b}+\frac{\gamma_{b}}{3}q_{i{,}_{i}}^{b}\right)+Q^{w}\left(\beta_{w}\partial_{t}Q^{w}+\frac{\gamma_{w}}{3}q_{i{,}_{i}}^{w}\right) \end{array}$$

as the entropy production, and the result of Remark A2 of the Appendix A is employed. We note that in the very general case $\gamma_{b}$ and $\gamma_{w}$ should be two scalar-valued functions depending on the whole set of the state variables, in principle.

In order to guarantee that the second law of thermodynamics is never violated, it is needed that $\sigma^{s}$ is never negative, whatever the thermodynamic process is. Sufficient conditions allowing this compliance, for example, may be the following relations

(10a) $$\begin{array}{c} \pi_{b}q_{i}^{b}=\alpha_{b}\partial_{t}q_{i}^{b}-\frac{\theta_{{,}_{i}}}{\theta^{2}}+\gamma_{b}Q_{ij{,}_{j}}^{b,0}+\gamma_{b}Q_{{,}_{i}}^{b} \end{array}$$

(10b) $$\begin{array}{c} \;\pi_{w}q_{i}^{w}=\alpha_{w}\partial_{t}q_{i}^{w}+\gamma_{w}Q_{ij{,}_{j}}^{w,0}+\gamma_{w}Q_{{,}_{i}}^{w} \end{array}$$

(10c) $$\begin{array}{c} \;\;\;\;\Pi_{b}Q_{ij}^{b,0}=\beta_{b}\partial_{t}Q_{ij}^{b,0}+\gamma_{b}q_{ij}^{b,\mathrm{sym},0} \end{array}$$

(10d) $$\begin{array}{c} \;\;\;\Pi_{w}Q_{ij}^{w,0}=\beta_{w}\partial_{t}Q_{ij}^{w,0}+\gamma_{w}q_{ij}^{w,\mathrm{sym},0} \end{array}$$

(10e) $$\begin{array}{c} \;\;\;\;\;\nu_{b}Q^{b}=\beta_{b}\partial_{t}Q^{b}+\frac{\gamma_{b}}{3}q_{i{,}_{i}}^{b} \end{array}$$

(10f) $$\begin{array}{c} \;\;\;\;\nu_{w}Q^{w}=\beta_{w}\partial_{t}Q^{w}+\frac{\gamma_{w}}{3}q_{i{,}_{i}}^{w} \end{array}$$

(10g) $$\begin{array}{c} \;\;\;\;\;\;\;\;\;\;q_{i{,}_{i}}^{w}=0 \end{array}$$

wherein $\pi_{b}$, $\pi_{w}$, $\Pi_{b}$, $\Pi_{w}$, $\nu_{b}$, and $\nu_{w}$ are suitable non-negative coefficients, that, in principle, are allowed to depend on the whole set of the state variables, similarly to the functions $\alpha_{b}$, $\alpha_{w}$, $\beta_{b}$, and $\beta_{w}$ introduced above.

If the sufficient conditions in Equation (10a–f) hold, we note that it should be tacitly understood that only linear relations between thermodynamic fluxes and forces are taken into account. Relations more complex than Equation (10a–f), in fact, should be introduced when dealing with nonlinear flux–force relations (see, for example, Chapter 2–Subsection 2.1.2 in Ref. [5] to this end). Although not properly the aim of this paper, we note that nonlinear flux–force relations would allow us to introduce in the theoretical model (2) genuinely nonlinear terms whose influence on the heat propagation at nanoscale, however, should deserve special attention since they may play a relevant role.

In closing this section we note that in the present approach Equation (10a–f) represent the evolution equations of the flux variables $q_{i}^{b}$, $q_{i}^{w}$, $Q_{ij}^{b,0}$, $Q_{ij}^{w,0}$, $Q^{b}$ and $Q^{w}$, respectively; in our approach all state variables have therefore their own evolution equations.

## 3. Physical Considerations: The Model Identification

It is tacitly understood that Equation (10a–f) will have relevance and may be employed for theoretical analyses as long as the different unknown coefficients appearing therein have been clearly identified on physical grounds. By comparing those equations with the equations which characterize some well-known theories of non-equilibrium Thermodynamics, in this section we relate $\alpha_{b}$, $\alpha_{w}$, $\beta_{b}$, $\beta_{w}$, $\gamma_{b}$, $\gamma_{w}$, $\pi_{b}$, $\pi_{w}$, $\Pi_{b}$, $\Pi_{w}$, $\nu_{b}$, and $\nu_{w}$ to some well-known physical quantities. The considerations below, merged with those of Section 2, can also be thought of as the theoretical macroscopic derivation of Equation (2a–g).

First of all, it is easy to recognize that the introduction of Equation (10g) into Equation (6) directly yields Equation (2a), if the usual thermodynamic relation $de=c_{v}d\theta$ is used.

Assume then a situation that is characterized by vanishing values of $Q_{i}^{b}$ so that Equation (10a) simplifies to the following:

$$-\alpha_{b}\partial_{t}q_{i}^{b}+\pi_{b}q_{i}^{b}=-\frac{\theta_{{,}_{i}}}{\theta^{2}}$$

In this case the evolution equation for the bulk heat flux is compatible with the Cattaneo’s equation [5,25]

$$\tau \partial_{t}q_{i}+q_{i}=-\lambda \theta_{{,}_{i}}$$

if, and only if, we make the following identifications:

(11a) $$\begin{array}{c} \alpha_{b}=-\frac{\tau_{b}}{\lambda \theta^{2}} \end{array}$$

(11b) $$\begin{array}{c} \pi_{b}=\frac{1}{\lambda \theta^{2}} \end{array}$$

Since $\alpha_{w}$ and $\alpha_{b}$, as well as $\pi_{w}$ and $\pi_{b}$, have the same physical dimensions, we may also infer the following:

(12a) $$\begin{array}{c} \alpha_{w}=-\frac{\tau_{w}}{\lambda \theta_{w}^{2}} \end{array}$$

(12b) $$\begin{array}{c} \pi_{w}=\frac{1}{\lambda \theta_{w}^{2}} \end{array}$$

wherein $\theta_{w}$ stands for the value of θ at the wall. Because in a first approach one may estimate $\tau_{b}=\frac{1}{f_{b}}$ and $\tau_{w}=\frac{1}{f_{w}}$, with $f_{b}$ and $f_{w}$ being the frequency of the bulk scatterings and of the phonon–boundary scattering, respectively, in principle it should be assumed that $\tau_{b}\neq \tau_{w}$. We, however, note that in Ref. [23] (see at the end of Section 2 therein) the relaxation time $\tau_{b}$ has been identified with the time lag between the application of a temperature gradient and the appearance of $q_{i}^{b}$, whereas $\tau_{w}$ has been meant therein as the time lag between the appearance of $q_{i}^{b}$ and the consequent appearance of $q_{i}^{w}$.

Once the above identifications have been made, we may further note that whenever the time variations of the higher-order flux $Q_{ij}^{b}$ are negligibly small (or, similarly, whenever $\beta_{b}\to 0$), instead, the coupling of Equation (10a,c,e) yield the following equation

$$\frac{\tau_{b}}{\lambda \theta^{2}}\partial_{t}q_{i}^{b}+\frac{1}{\lambda \theta^{2}}q_{i}^{b}=-\frac{\theta_{{,}_{i}}}{\theta^{2}}+\gamma_{b}^{2}\left[\frac{1}{2\Pi_{b}}q_{i{,}_{jj}}^{b}+\frac{1}{3}\left(\frac{1}{2\Pi_{b}}+\frac{1}{\nu_{b}}\right)q_{j{,}_{ji}}^{b}\right]$$

which is compatible with (i.e., similar to) the Guyer–Krumhansl’s equation [5,26,27]

$$\tau \partial_{t}q_{i}+q_{i}=-\lambda \theta_{{,}_{i}}+\ell^{2}\left(q_{i{,}_{jj}}+2q_{j{,}_{ji}}\right)$$

if, and only if,

(13a) $$\begin{array}{c} \gamma_{b}=-\frac{\tau_{b}}{\lambda \theta^{2}} \end{array}$$

(13b) $$\begin{array}{c} \Pi_{b}=\frac{\tau_{b}^{2}}{2\ell^{2}\lambda \theta^{2}} \end{array}$$

(13c) $$\begin{array}{c} \nu_{b}=\frac{\tau_{b}^{2}}{5\ell^{2}\lambda \theta^{2}} \end{array}$$

Since $\gamma_{w}$ and $\gamma_{b}$, $\Pi_{b}$, and $\Pi_{w}$, and $\nu_{w}$ and $\nu_{b}$, have the same physical dimensions, we are also allowed to assume

(14a) $$\begin{array}{c} \gamma_{w}=-\frac{\tau_{w}}{\lambda \theta_{w}^{2}} \end{array}$$

(14b) $$\begin{array}{c} \Pi_{w}=\frac{\tau_{w}^{2}}{2\xi^{2}\ell^{2}\lambda \theta_{w}^{2}} \end{array}$$

(14c) $$\begin{array}{c} \nu_{w}=\frac{\tau_{w}^{2}}{5\xi^{2}\ell^{2}\lambda \theta_{w}^{2}} \end{array}$$

For dimensional reasons we may assume the following further relations:

(15a) $$\begin{array}{c} \beta_{b}=-\frac{\tau_{b}^{2}\tau_{b}^{★}}{2\ell^{2}\lambda \theta^{2}} \end{array}$$

(15b) $$\begin{array}{c} \beta_{w}=-\frac{\tau_{w}^{2}\tau_{w}^{★}}{2\xi^{2}\ell^{2}\lambda \theta_{w}^{2}} \end{array}$$

As a consequence of the identifications above, $\alpha_{b}$, $\alpha_{w}$, $\beta_{b}$, and $\beta_{w}$ are negative scalar-valued functions: this guarantees that *s* has a maximum at the equilibrium, according to Equation (3).

Recalling that the present analysis is carried on in *close-to-equilibrium* situations (i.e., when the different material functions can be treated as constants), all coefficients appearing in Equation (10a–f) become constants whenever the non-equilibrium-temperature approximation [5,28] holds, namely, when $\frac{1}{\theta^{2}}\approx \frac{1}{\theta_{w}^{2}}\approx \frac{1}{\theta_{\mathrm{eq}}^{2}}$, with $\theta_{\mathrm{eq}}$ being the (constant value of the) local-equilibrium temperature. In this special case, in particular, from Equation (3), the following constitutive equation for *s* arises:

(16) $$s=s_{\mathrm{eq}}\left(e\right)-\frac{\tau_{b}}{2\lambda \theta^{2}}q_{i}^{b}q_{i}^{b}-\frac{\tau_{w}}{2\lambda \theta_{w}^{2}}q_{i}^{w}q_{i}^{w}-\frac{\tau_{b}^{2}\tau_{b}^{★}}{4\ell^{2}\lambda \theta^{2}}Q_{ij}^{b}Q_{ij}^{b}-\frac{\tau_{w}^{2}\tau_{w}^{★}}{4\xi^{2}\ell^{2}\lambda \theta_{w}^{2}}Q_{ij}^{w}Q_{ij}^{w}$$

Whereas the choice of the signs’ coefficients $\alpha_{b}$, $\alpha_{w}$, $\beta_{b}$, and $\beta_{w}$ is strictly related to the principle of maximum entropy at equilibrium, we are not presently aware of an experimental reason for the choice of the signs of the coefficients $\gamma_{b}$ and $\gamma_{w}$, instead. We here suppose that those coefficients are negative since in this way the form of the constitutive equation of $J_{i}^{s}$, that is, Equation (3), reads as follows

(17) $$J_{i}^{s}=\frac{q_{i}^{b}}{\theta }-\frac{\tau_{b}}{\lambda \theta^{2}}Q_{ij}^{b}q_{j}^{b}-\frac{\tau_{w}}{\lambda \theta_{w}^{2}}Q_{ij}^{w}q_{j}^{w}$$

and is compatible with the form of the specific-entropy flux found in EIT (see, for example, Chapter 9–Subsection 9.3.2 in Ref. [5]).

If the identifications of the different coefficients in Equations (11a,b)–(15a,b) hold, then the general relations in Equation (10a–f) directly yield the particular model described by Equation (2a–g), once Equation (6) is also taken into account.

## 4. Heat Waves

The theoretical model in Equation (2a–g) is hyperbolic, namely, it predicts that (small-amplitude) thermal disturbances propagate with a finite speed. To clearly show this feature of Equation (2a–g), in the present section we therefore study the propagation of thermal signals (or, alternatively said, temperature waves). In doing this we here employ the mathematical tool of a planar surface $S\left(t\right)$ which propagates along the system with a non-vanishing velocity *V* (whose value is unknown a priori) and across which the time and/or the spatial derivatives of the state variables suffer finite jumps (i.e., at most finite discontinuities), whereas the state variables are continuous everywhere in the system [29,30,31,32].

Recalling that planar surfaces are also strictly related to Hadamard’s lemma (see, for example, Appendix A.b in Ref. [32]) in such a way that for a generic state variable $\psi \left(x_{i},t\right)$ one has

(18) $$〚\partial_{t}\psi 〛=-V〚n_{i}\psi_{,i}〛$$

with 〚·〛 being the amplitude of the finite jump across S of the indicated quantity. Then by taking the jumps of Equation (2a–g) we straightforwardly have the following linear system:

(19a) $$\begin{array}{c} \;\;\;\;\;\;-V\Theta +\frac{n_{i}}{c_{v}}\Gamma_{i}^{b}=0 \end{array}$$

(19b) $$\begin{array}{c} -V\Gamma_{i}^{b}+\frac{\lambda n_{i}}{\tau_{b}}\Theta +n_{j}\Lambda_{ij}^{b}+n_{i}\Pi^{b}=0 \end{array}$$

(19c) $$\begin{array}{c} \;\;\;-V\Gamma_{i}^{w}+n_{j}\Lambda_{ij}^{w}+n_{i}\Pi^{w}=0 \end{array}$$

(19d) $$\begin{array}{c} \;\;\;\;-V\Lambda_{ij}^{b}+\frac{4\ell^{2}n_{j}}{3\tau_{b}\tau_{b}^{★}}\Gamma_{i}^{b}=0 \end{array}$$

(19e) $$\begin{array}{c} \;\;\;-V\Pi^{b}+\frac{5\ell^{2}n_{i}}{3\tau_{b}\tau_{b}^{★}}\Gamma_{i}^{b}=0 \end{array}$$

(19f) $$\begin{array}{c} \;\;\;-V\Lambda_{ij}^{w}+\frac{2\xi^{2}\ell^{2}n_{j}}{\tau_{w}\tau_{w}^{★}}\Gamma_{i}^{w}=0 \end{array}$$

(19g) $$\begin{array}{c} \;\;\;\;\;\;\;\;-V\Pi^{w}=0 \end{array}$$

wherein $\Theta :=〚n^{i}\theta_{{,}_{i}}〛$, $\Gamma_{i}^{b}:=〚n^{j}q_{i{,}_{j}}^{b}〛$, $\Gamma_{i}^{w}:=〚n^{j}q_{i{,}_{j}}^{w}〛$, $\Lambda_{ij}^{b}:=〚n^{k}Q_{ij{,}_{k}}^{b,0}〛$, $\Lambda_{ij}^{w}:=〚n^{k}Q_{ij{,}_{k}}^{w,0}〛$, $\Pi^{b}:=〚n^{i}Q_{{,}_{i}}^{b}〛$ e $\Pi^{w}:=〚n^{i}Q_{{,}_{i}}^{w}〛$. We note that by the way in obtaining Equation (19a–g) we’ve made use of the relations about $S\left(t\right)$ contained in Ref. [32] (see Appendix A.b therein).

In order that the linear system (19a–g) does not admit the only trivial solution, i.e., avoiding the meaningless case in which all the jumps’ amplitudes are null, straightforward calculations yield that the following relation has to be fulfilled:

(20) $$\left(V^{2}-\frac{\lambda }{c_{v}\tau_{b}}-\frac{3\ell^{2}}{\tau_{b}\tau_{b}^{★}}\right)\left(V^{2}-\frac{2\xi^{2}\ell^{2}}{\tau_{w}\tau_{w}^{★}}\right)=0$$

As a consequence of Equation (20), in principle we may claim that the model in Equation (2a–g) prescribes that a thermal wave will propagate in the bulk with a speed $V^{b}$ that is different with respect to the speed of propagation $V^{w}$ in the Knudsen layer because one has

(21a) $$\begin{array}{c} V^{b}=\sqrt{\frac{\lambda }{c_{v}\tau_{b}}+\frac{3\ell^{2}}{\tau_{b}\tau_{b}^{★}}} \end{array}$$

(21b) $$\begin{array}{c} \;\;V^{w}=\sqrt{\frac{2\xi^{2}\ell^{2}}{\tau_{w}\tau_{w}^{★}}} \end{array}$$

From a practical point of view, the above result can be interpreted in the following way. The presence of the Knudsen layer yields that a thermal perturbation in a point of the system will generate the propagation of two different waves: one in the bulk with a speed $V^{b}$ and the other in the Knudsen layer with a speed $V^{w}$. Because in principle it seems logical that $\tau_{b}$ and $\tau_{w}$ have the same order of magnitude, as well as $\tau_{b}^{★}$ and $\tau_{w}^{★}$, then from Equation (21a,b) we have $V^{w}<V^{b}$: from the results above we may infer that the Knudsen layer slows down the propagation of thermal waves. We are currently not aware of experimental evidence proving this result, which, however, seems a logical and direct consequence of the main difference between Equation (2b,c) commented on in Section 2 of Ref. [23] and its possible physical explanation: the presence of the temperature gradient in the former equation suggests that it is the driving force (or, alternatively said, the cause) of the propagation of the bulk heat flux, which in turn is then the driving force (or, alternatively said, the cause) of the propagation of the wall heat flux. In this new approach to the thermal conductivity at nanoscale, as a consequence, in the bulk of the system the speed of propagation of thermal waves is both related to the temperature gradient (responsible for the rate $\frac{\lambda }{c_{v}\tau_{b}}$ in Equation (21a)) and to non-local effects (responsible for the rate $\frac{3\ell^{2}}{\tau_{b}\tau_{b}^{★}}$ in Equation (21a)); in the Knudsen layer, instead, the speed of propagation of thermal waves is only related to nonlinear effects, which are responsible for the sole rate $\frac{2\xi^{2}\ell^{2}}{\tau_{w}\tau_{w}^{★}}$ in Equation (21b).

## 5. Final Comments

The right modeling of the heat transport at nanoscale is an important goal towards a better description of the energy flow in materials, as well as for many modern technological applications. Nowadays it is well-known that the thermal transport at the nanoscale is fundamentally different from that at the macroscale, wherein the Fourier law is usually employed.

Whereas several works have shown that the Fourier law dramatically overpredicts the rate of heat dissipation from heat sources that are characterized by dimensions smaller than the mean-free path of the heat carriers, from the theoretical point of view it is also well-known that the continuum model of Fourier’s law of heat conduction violates the relativity theory because it complies with instantaneous thermal responses.

In order to go beyond the Fourier law, in this paper we therefore discussed Equation (2a–g), i.e., a new theoretical model describing the heat transfer at nanoscale in a rigid body that introduces the concept of the Knudsen layer into non-equilibrium Thermodynamics. Its concept, borrowed from gas dynamics, in essence describes the region near the system’s boundary wherein phonon–wall interactions dominate over bulk scattering events in such a way that Equation (2a–g) have the great advantage of deepening the role played by the phonon–boundary scattering. This could be interesting because understanding the influence of that scattering mechanism could be essential for an accurate modeling of heat transport in nanostructures.

Being in agreement with the second law, the theoretical model described by Equation (2a–g) is thermodynamically consistent, as it has been pointed out in Section 2 and Section 3. That model also allows us to avoid the paradox of infinite speed of propagation of heat waves; the analysis performed in Section 4, moreover, quantifies how phonon–boundary scattering may influence the thermal-wave propagation, especially near the boundaries of the system. The dual-wave prediction of Section 4 is new and physically insightful: it predicts that heat disturbances at nanoscale do not spread uniformly, but rather through coupled bulk and boundary dynamics. Such predictions could guide experimental detection of second sound in nanosystems.

Because we currently have no evidence of the results contained in Section 4, we close this paper by observing that they should be therefore only be meant as a theoretical prediction. We, however, feel that the above possible generation of two different thermal waves should deserve a deeper scrutiny. To support this claim, we note that in Ref. [28], for example, the theoretical second-harmonic generation of thermal waves has been theoretically investigated by means of nonlinear relations. The accounting for the Knudsen layer (i.e., deepening the influence of the phonon–boundary scattering on the thermal-wave propagation) in a linear theory seems to yield results that would be otherwise only strictly related to nonlinear effects.

We finally note that in Ref. [33] the author assumes that phonons may propagate over short distances also in liquids, supposed as dual systems made by islands of solids fluctuating in the amorphous classical structure, and proposes therein that such regular motion may be the cause of the phenomenon of shear strain observed in liquids that generates a temperature distribution in the liquid matrix. Interestingly, in Ref. [34], the same dual model is also used to investigate how heat propagates in liquids. It would be interesting to investigate the possible influence of the Knudsen layer in the particular case of the aforementioned solid–liquid interfaces. In this case the two different speeds of propagation carried out in Section 4 could be related to the velocity of propagation of phonons within the solid islands and at their interface with amorphous liquid in the dual model liquids. 

## Data Availability

No new data were created or analyzed in this study. Data sharing is not applicable to this article.

## Appendix A

**Remark** **A1.**
*For the sake of clarity, in the table below we list the physical quantities involved in the present paper with their own physical dimensions.*

**Symbol**	**Meaning**	**Unit of Measurement**
θ	non-equilibrium temperature	K
$\theta_{w}$	value of θ at the wall	K
$\theta_{\mathrm{eq}}$	local-equilibrium temperature	K
*e*	internal energy per unit volume	${\mathrm{Jm}}^{-3}$
$q_{i}$	local heat flux	${\mathrm{Wm}}^{-2}$
$q_{i}^{b}$	bulk heat flux	${\mathrm{Wm}}^{-2}$
$Q_{ij}^{b}$	flux of $q_{i}^{b}$	${\mathrm{Wm}}^{-1}s^{-1}$
$Q_{ij}^{b,0}$	deviatoric (traceless) part of $Q_{ij}^{b}$	${\mathrm{Wm}}^{-1}s^{-1}$
$Q^{b}\delta_{ij}$	spherical part of $Q_{ij}^{b}$	${\mathrm{Wm}}^{-1}s^{-1}$
$q_{i}^{w}$	wall heat flux	${\mathrm{Wm}}^{-2}$
$Q_{ij}^{w}$	flux of $q_{i}^{w}$	${\mathrm{Wm}}^{-1}s^{-1}$
$Q_{ij}^{w,0}$	deviatoric (traceless) part of $Q_{ij}^{w}$	${\mathrm{Wm}}^{-1}s^{-1}$
$Q^{w}\delta_{ij}$	spherical part of $Q_{ij}^{w}$	${\mathrm{Wm}}^{-1}s^{-1}$
$\tau_{b}$	relaxation time of $q_{i}^{b}$	s
$\tau_{w}$	relaxation time of $q_{i}^{w}$	s
$\tau_{b}^{★}$	relaxation time of $Q_{ij}^{b}$	s
$\tau_{w}^{★}$	relaxation time of $Q_{ij}^{w}$	s
λ	thermal conductivity	${\mathrm{Wm}}^{-1}K^{-1}$
*ℓ*	phonon mean-free path in the bulk	m
$c_{v}$	specific heat per unit volume at constant volume	${\mathrm{Jm}}^{-3}K^{-1}$
*s*	entropy per unit volume	${\mathrm{Jm}}^{-3}K^{-1}$
$J_{i}^{s}$	entropy flux per unit volume	${\mathrm{Wm}}^{-2}K^{-1}$
$\sigma^{s}$	entropy production per unit volume	${\mathrm{Wm}}^{-3}K^{-1}$
*V*	thermal-wave speed	${\mathrm{ms}}^{-1}$
$V^{b}$	thermal-wave speed in the bulk	${\mathrm{ms}}^{-1}$
$V^{w}$	thermal-wave speed in the Knudsen layer	${\mathrm{ms}}^{-1}$

**Remark** **A2.**
*Let $S_{ij}$ be a symmetric second-order tensor and $W_{ij}$ be a skew-symmetric second-order tensor. Since the following is obtained:*

$$S_{ij}W_{ij}=-S_{ji}W_{ji}=-S_{ij}W_{ij}$$

*then the scalar product between those tensors vanishes, i.e., $S_{ij}W_{ij}=0$.*
